# Identification of the Light-Harvesting Chlorophyll a/b Binding Protein Gene Family in Peach (*Prunus persica* L.) and Their Expression under Drought Stress

**DOI:** 10.3390/genes14071475

**Published:** 2023-07-19

**Authors:** Li Wang, Jia Wei, Xingyun Shi, Weihong Qian, Jan Mehmood, Yiming Yin, Huijuan Jia

**Affiliations:** 1 Huzhou Academy of Agricultural Sciences, Huzhou 313000, China; wangli3852@163.com (L.W.); shixingyunlove@163.com (X.S.); dder_chen@163.com (W.Q.); 2 College of Agriculture and Biotechnology, Zhejiang University, Hangzhou 310058, China; weijia_2008_china@163.com (J.W.); mehmoodjan89@zju.edu.cn (J.M.)

**Keywords:** light-harvesting chlorophyll a/b binding protein, peach, gene family, drought stress, *Prunus persica*

## Abstract

In higher plants, light-harvesting chlorophyll a/b binding (Lhc) proteins play a vital role in photosynthetic processes and are widely involved in the regulation of plant growth, development, and response to abiotic stress. However, the *Lhc* gene family has not been well identified in peaches (*Prunus persica* L.). In this study, 19 *PpLhc* genes were identified in the peach genome database, which were unevenly distributed on all chromosomes. Phylogenetic analysis demonstrated that PpLhc proteins could be divided into three major subfamilies, each of whose members had different exon–intron structures but shared similar conserved motifs. A total of 17 different kinds of cis-regulatory elements were identified in the promoter regions of all *PpLhc* genes, which could be classified into three categories: plant growth and development, stress response, and phytohormone response. In addition, transcriptomic data analysis and RT-qPCR results revealed that the expression profiles of some *PpLhc* genes changed under drought treatment, suggesting the crucial roles of *Lhc* genes in the regulation of plant tolerance to drought stress. Taken together, these findings will provide valuable information for future functional studies of *PpLhc* genes, especially in response to drought stress.

## 1. Introduction

Photosynthesis is the basis for the survival of most plants [1]. Light capture is the first and most important event in oxygenic photosynthesis, which is mediated by the light capture Lhc protein [2]. In plant cells, Lhc family proteins are encoded in the nucleus, translated in the cytosol, and finally introduced into chloroplasts [3]. The Lhc protein is mainly located on the chloroplast thylakoid membrane and combines with pigment molecules to form a pigment–protein complex that is responsible for harvesting and transmitting solar energy during photosynthesis. The Lhc protein possesses three transmembrane helices and a highly conserved chlorophyll a/b binding domain [4]. The Lhc family can be mainly divided into two subfamilies, known as Lhca and Lhcb, which are related to the light-harvesting complexes LHCI and LHCII, respectively [5,6,7]. Based on phylogenetic analysis, Lhca could be further divided into six groups: Lhca1–6, and Lhcb could be divided into eight groups: Lhcb1–8, where Lhcb8 only exists in eudicotyledons [8,9]. Based on crystal structure analyses, LHCI in PSI is mainly composed of Lhca1, -2, -3, and -4, which are tightly integrated within the core complex, and LHCII in PSII is mainly comprised of Lhcb1–6, where Lhcb1–3 form the main trimer and Lhcb4/CP29, Lhcb5/CP26, and Lhcb6/CP24 are in monomeric form [10,11,12,13,14].

In plants, Lhc family members play important roles in regulating growth and development. In Arabidopsis, *Lhcb* genes participate in seed germination and post-germination growth [15]. Downregulation of Arabidopsis *Lhcb1* leads to slightly smaller and paler leaves with a lower content of chlorophyll [3]. Members of the Lhc family in kiwifruit and cotton have been proven to be involved in the synthesis of chlorophyll a [16,17]. Transient overexpression of *AcLhcb3.1*/*3.2* in tobacco leaves significantly increases the content of chlorophyll a [16], and VIGS-mediated silencing of *GhLhcb2.3* results in a significant decrease in chlorophyll a content in cotton leaves [17]. In *Sedum alfredii*, overexpression of *SaLhcb2* increases the biomass of shoots and roots [18]. In barley, some single nucleotide polymorphisms (SNPs) in *Lhcb1* have been found to be significantly associated with various agronomic traits, such as plant height and leaf color [19].

Lhc proteins also regulate plant responses to abiotic stress. In rice seedlings, Lhca1–4 proteins are significantly reduced under Fe deficiency, resulting in decreased chlorophyll and photosynthetic efficiency [20]. In celery, the expression of Lhcb1 is increased under heat, cold, salt, and drought stress [21]. It has been reported that *AtLhcb1–6* play a positive role in plant tolerance to drought stress in Arabidopsis. Downregulation or disruption of any member of these genes reduces the responsiveness of stomatal movement to ABA, and consequently leads to decreased tolerance to drought stress [22]. Furthermore, formaldehyde stress in Arabidopsis also impacts the expression of *Lhcb2.1*, *Lhcb3*, and *Lhca4* [23]. In tobacco, overexpression of the *LeLhcb2* gene enhances plant tolerance to chilling stress and alleviates photooxidation of PSII [24]. Overexpression of *SaLhcb2* in tobacco increases the cadmium absorption capacity of transgenic plants [18]. Previous research has shown that the *Lhc* genes in apple show different expression patterns under drought stress. Overexpressing *MdLhcb4.3* enhances the resistance of apple callus and transgenic Arabidopsis to drought and osmotic stress [25]. A recent study demonstrates that silencing of *TaLHC86* by BSMV-VIGS seriously affects the photosynthetic rate and electron transport, and reduces the salt tolerance in wheat [26]. These findings indicate that Lhc proteins play a vital role in the regulation of plant tolerance to various stresses.

The peach belongs to the Rosaceae family and is a highly popular and economically important fruit crop worldwide. However, drought stress poses a significant threat to the production and economic viability of peach fruit. With the completion and release of the whole genome sequence of the peach, the identification of key candidate gene families has become more convenient. The *Lhc* gene family is known to play crucial roles in the regulation of various physiological processes and responses to different abiotic stressors. Despite this, a comprehensive analysis of the peach *Lhc* (designated *PpLhc*) gene family has yet to be conducted. Therefore, the aim of this study was to identify the *Lhc* gene family members in the peach and investigate their functions under drought stress. In this study, genome-wide identification of *PpLhc* members was carried out by analyzing the peach genome database. Gene distributions, physical and chemical properties, phylogenetic relationships, exon–intron architectures, conserved motifs, predicted subcellular locations, and cis-regulatory elements were analyzed. In addition, the transcript levels of *PpLhc* family members under drought treatment were analyzed using the relevant RNA-seq data and verified by real-time quantitative PCR (RT-qPCR). Our results lay the foundation for future functional study of the *PpLhc* genes, more importantly, for the use of *PpLhc* genes to improve drought tolerance in peaches.

## 2. Materials and Methods

### 2.1. Genome-Wide Identification of PpLhc Genes

The peach genome and annotation files were obtained from the Genome Database for Rosaceae (GDR) (https://www.rosaceae.org/species/prunus_persica/genome_v2.0.a1, accessed on 13 December 2022). To identify the Lhc proteins of peaches, the domain of Lhc-specific hidden Markov models (HMMs) PF00504 was downloaded from the Pfam database (http://pfam.xfam.org/, accessed on 16 June 2023) and used as the query to search the potential peach Lhc members in the peach genome using hmmsearch (HMMER 3.0). Next, a BLASTP search against the potential peach Lhc members was performed by aligning the sequences of the 21 AtLhc protein sequences downloaded from the Arabidopsis Information Resource (http://www.arabidopsis.org, accessed on 16 June 2023), with the E-value cut-off of the alignment set as e-05. PfamScan (version 1.6) and the SMART were further used to determine the sequences possessing the PF00504 domain as the final Lhc sequences. To distinguish the candidate *PpLhc* genes, they were named according to their phylogenetic relationship with that of Arabidopsis.

### 2.2. Analysis of Chromosome Distribution and Physicochemical Properties of PpLhc Proteins

The annotated files and the length information for the peach chromosome downloaded from GDR were used to extract the chromosome distribution information of *PpLhc* gene family members, and Tbtools V1.098 software [27] was used to draw the chromosome distribution map, with the *Lhc* family members labeled on the corresponding chromosomes. The physical and chemical characteristics of PpLhc proteins were calculated in the ProtParam tool (https://web.expasy.org/protparam/, accessed on 13 December 2022) [28].

### 2.3. Phylogenetic Relationships Analysis

The phylogenetic analysis was conducted using MEGA 7.0 [29]. The full-length amino acid sequences of Arabidopsis and peach Lhc proteins were aligned using ClustalW [30], and the resulting alignment was used to construct the maximum likelihood (ML) phylogenetic tree, with a bootstrap test of 1000 replicates. The tree was finally displayed using Interactive Tree of Life (iTOL v5) [31].

### 2.4. Gene Structure and Protein Motif Analysis

The exon–intron organization information for *PpLhc* genes was retrieved from the peach genome annotation file and visualized using the online Gene Structure Display Server 2.0 (http://gsds.gao-lab.org/, accessed on 16 June 2023) [32]. The conserved motifs of the PpLhc amino acid sequences were predicted using MEME Suite [33]. The motif value was set to 15 for the search, and other parameters were kept as defaults. The structure diagrams of the conserved protein motifs were constructed by Tbtools V1.098.

### 2.5. Prediction of Subcellular Localization and Transmembrane Domain

The subcellular localizations of the peach Lhc proteins were predicted by SoftBerry (http://www.softberry.com/, accessed on 16 June 2023), Predator (https://urgi.versailles.inra.fr/predotar/predotar.html, accessed on 13 December 2022), and WoLF PSORT (http://www.genscript.com/psort/wolf_psort.html, accessed on 13 December 2022). The transmembrane domains were predicted by the online program DeepTMHMM (https://dtu.biolib.com/DeepTMHMM, accessed on 13 December 2022).

### 2.6. Collinearity and Promoter Element Analysis

Genome annotation files of peach (Prunus persica Genome v2.0.a1), apple (https://www.rosaceae.org/species/malus/malus_x_domestica/genome_GDDH13_v1.1, accessed on 13 December 2022) and Arabidopsis (TAIR10.1) were downloaded from the databases GDR and TAIR, respectively. Genome-wide collinearity analysis among Arabidopsis, peach, and apple was conducted using MCScanX software [34] and visualized using TBtools software. To analyze the promoter element, the 2 kb upstream sequences of coding regions of each *PpLhc* gene obtained from the *Prunus persica* peach genome database were submitted to PlantCARE (http://bioinformatics.psb.ugent.be/webtools/plantcare/html/, accessed on 13 December 2022) [35] for the prediction of promoter elements, and then the distribution map of promoter elements was drawn using Tbtools V1.098 software [27].

### 2.7. RNA-Seq Data Analysis

The raw RNA-seq data of peach fruit flesh samples under different drought treatment times were downloaded from the Sequence Read Archive (SRA) database of the National Center for Biotechnology Information (NCBI) with accession number PRJNA323761. The FastQC tool was used to check the quality of the raw sequencing data [36], and Trimmomatic [37] was used to trim the adapter and low-quality bases (phred score < 33). The clean reads from each sample were mapped into the peach genome using Hisat2 version 2.2.1 [38] with default parameters. Further transcript assembly and quantification of the read alignments were performed with Stringtie [39]. Transcripts per kilobase million (TPM) were used to quantify gene expression levels. The heatmap of gene expression levels was constructed using Tbtools V1.098 software [27].

### 2.8. Plant Materials and Treatments

One-year-old peach cv. “Gengcunyangtao” seedlings were maintained at the experimental orchard of the Huzhou Academy of Agricultural Sciences (Wuxing District, Huzhou, China). For PEG treatments, the soil on the roots was carefully removed, and then the seedlings were treated with a 20% (*w*/*v*) PEG-6000 solution. Roots and leaves were sampled at 0 h, 3 h, 6 h, and 12 h after treatment. Each sample consisted of three biological replicates, and each contained at least 2 leaves collected from 1 seedling.

### 2.9. RNA Extraction and RT-qPCR

All fresh samples were frozen in liquid nitrogen and homogenized for RNA isolation with TRIzol reagent (Invitrogen, Waltham, MA, USA) according to the manufacturer’s protocol. The first-stranded cDNA was produced from 1 μg of total RNA using a MonScriptTM RTIII Super Mix with dsDNase (Monad Biotech, Wuhan, China), following the manufacturer’s instructions. Reactions were performed with SYBR Premix on a LightCycler480 II Real-Time PCR machine (Roche) with the following procedure: 1 min at 95 °C, and then 40 cycles of 10 s at 95 °C, 20 s at 60 °C, and 20 s at 72 °C. The primers for RT-qPCR analysis of the 5 *PpLhc* genes were designed using Primer 5.0 software. The *β-actin* gene was used as an internal control [40]. For each sample, at least three biological replicates were analyzed. The relative expression level of each gene is calculated using the 2^−ΔΔCT^ normalized expression method [41]. All primers are shown in Table S1.

### 2.10. Data Analysis

Statistical analyses of the data were performed with SPSS statistical software (version 20). A two-tailed Student’s *t*-test was conducted to determine significant differences at *p* < 0.05. All data analyses were based on three independent replicates. Graphs were prepared using Excel, and image processing was conducted with Photoshop CS6.

## 3. Results

### 3.1. Genome-Wide Identification and Characteristics of PpLhc Genes

**Figure 1:**
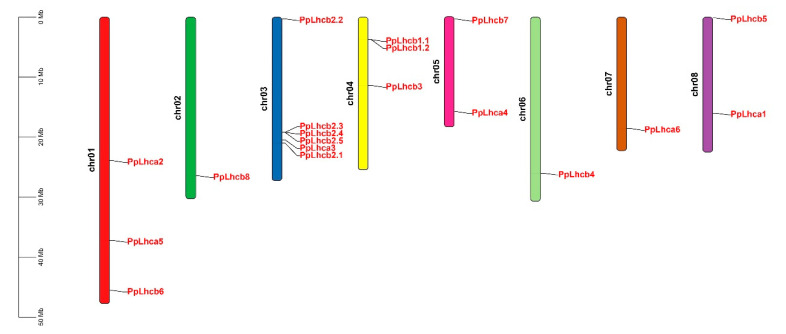
The location of *PpLhc* genes on the peach chromosome. Chromosomes are indicated with long colored bars, and chromosome numbers are shown at the left of each chromosome.

An HMM search was first used to obtain a complete list of peach Lhcs, which were then used for a BLASTP search using the 21 known Arabidopsis Lhc proteins as queries; thus, 19 *PpLhc* genes were obtained in the peach genome (Table 1). These *PpLhc* genes were unevenly distributed on all 8 chromosomes (Figure 1). Chr. 3 had the highest number of *PpLhc* genes with 6, followed by Chr. 1, Chr. 4, Chr. 5, and Chr. 8 with 3, 3, 2, and 2 PpLhc genes, respectively, and the other 3 chromosomes possessed only 1 *PpLhc* gene. The physical and chemical properties of PpLhc proteins were also investigated (Table 1). The predicted protein length ranged from 208 (PpLhcb2.5) to 327 (PpLhcb7), with an average amino acid number of 268. The predicted molecular weights ranged from 22.59 kDa (PpLhcb2.5) to 36.16 kDa (PbrLhcb8), and the predicted theoretical isoelectric points (pI) ranged from 5.11 (PpLhcb3) to 7.85 (PpLhca3). Moreover, the analysis of the GRAVY index revealed that all peach Lhc proteins were hydrophilic (less than or close to 0). The aliphatic index analysis also confirmed that all peach Lhc proteins had low values, below 100, except for PpLhcb7 (101.1), which further supported the predication that these PpLhc proteins are hydrophilic (Table 1).

### 3.2. Phylogenetic Relationship of Peach Lhc Proteins

**Figure 2:**
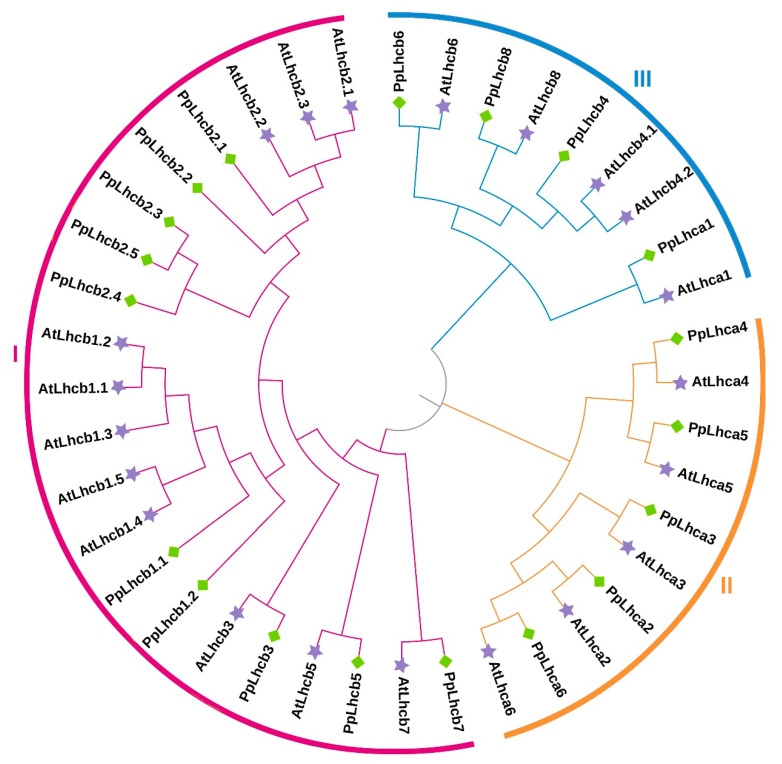
Phylogenetic analysis of Lhc proteins among peach and Arabidopsis. Purple stars represent AtLhc proteins, and green rhombuses represent PpLhc proteins.

To analyze the phylogenetic relationship between different PpLhcs, we constructed a phylogenetic tree based on the alignment of the Lhc protein sequences from all the Lhc family members in peach and Arabidopsis. It showed that PpLhc proteins can be classified into three subfamilies (I–III), which included 4, 5, and 10 PpLhc family members, respectively (Figure 2). It is worth mentioning that PpLhcb6, PpLhcb8, and PpLhca1 were strictly clustered with AtLhcb6, AtLhcb8, and AtLhca1 in subfamily I. Similarly, PpLhca2–6 in Group II exhibited close clustering with AtLhca2–6, respectively. Moreover, PpLhcb3, PpLhcb5, and PpLhcb7 in Group III exhibited close clustering with AtLhcb3, AtLhcb5, and AtLhcb7 of Arabidopsis, respectively (Figure 2), suggesting that the biological functions of these genes were similar.

### 3.3. Gene Structure and Protein Motif Analyses of PpLhc Members

To investigate the genetic structural diversity of *PpLhc* genes, we analyzed their structural characteristics using GSDS 2.0. The evolutionary analysis also classified the 19 *PpLhc* genes into three subfamilies. However, there were significant differences in the exon/intron structures of the different *PpLhc* genes, whether among the three subfamilies or in the same subfamily. Among these, five genes contained only one exon, all belonging to subfamily I. Additionally, there were four *PpLhc* genes with two exons. However, *PpLhca5*, *PpLhcb5*, and *PpLhcb7* each had the largest number of exons, at six. These results implied that the exons of the *PpLhc* gene family have undergone losses or increases during evolution.

**Figure 3:**
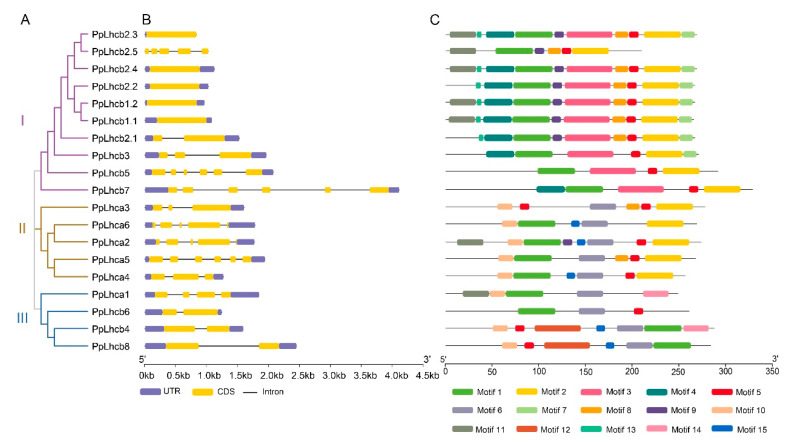
Phylogeny, gene structure, and conserved motif analysis of PpLhcs: (**A**) phylogenetic tree analysis of 19 PpLhc members; (**B**) schematic diagram of the exon–intron distributions of *PpLhc* genes. UTRs are represented by purple boxes, exons are represented by orange boxes, and introns are represented by single lines between the two exons; (**C**) schematic diagram of the conserved protein motifs of PpLhc members. Motifs 1 to 15 are marked with different colored boxes.

We used the MEME suite to analyze the conserved motifs in PpLhc proteins (Figure 3, Table S2). It was shown that motifs 1, 2, and 5 were present in most of the identified PpLhc proteins. Additionally, different subfamily members contained different conserved motifs, while members of the same subfamily tended to have similar ones. For example, all subfamily I members contain the motifs 1, 2, 3, and 5. Moreover, some motifs were specifically identified in different subfamilies. Motifs 12 and 14 were specifically in the proteins of subfamily III, and motifs 4, 7, and 13 were specifically possessed by subfamily I members, which, however, did not harbor motifs 6 and 15. Therefore, the results of the conserved motif analysis further supported the evolution study of the *PpLhc* gene family.

### 3.4. Subcellular Localization and Transmembrane Prediction of PpLhc Proteins

Chloroplast localization of the Lhc protein is vital for its biological function. Previous studies have shown that Lhc proteins are mainly targeted in the chloroplast. We first used the SoftBerry database to predict the subcellular localization of PpLhc proteins. The result showed that all PpLhc proteins were located in chloroplasts (Table 2). The prediction was further checked using Predotar and WoLf PSORT. Most of the PpLhc proteins were predicted to target the chloroplast by both online tools, except PpLhca5, PpLhcb7, and PpLhcb8, whose chloroplast localizations were supported only by one web server (Table 2). The Lhc protein usually possesses three transmembrane helices [4], and the membrane-binding features predicted by SoftBerry prompted us to predict whether these proteins have transmembrane (TM) domains. Transmembrane prediction by DeepTMHMM suggested that most peach Lhc family proteins possessed three TM domains, except PpLhca2 and PpLhcb7, which had only one predicted TM domain.

### 3.5. Collinearity Analysis of PpLhc Genes

**Figure 4:**
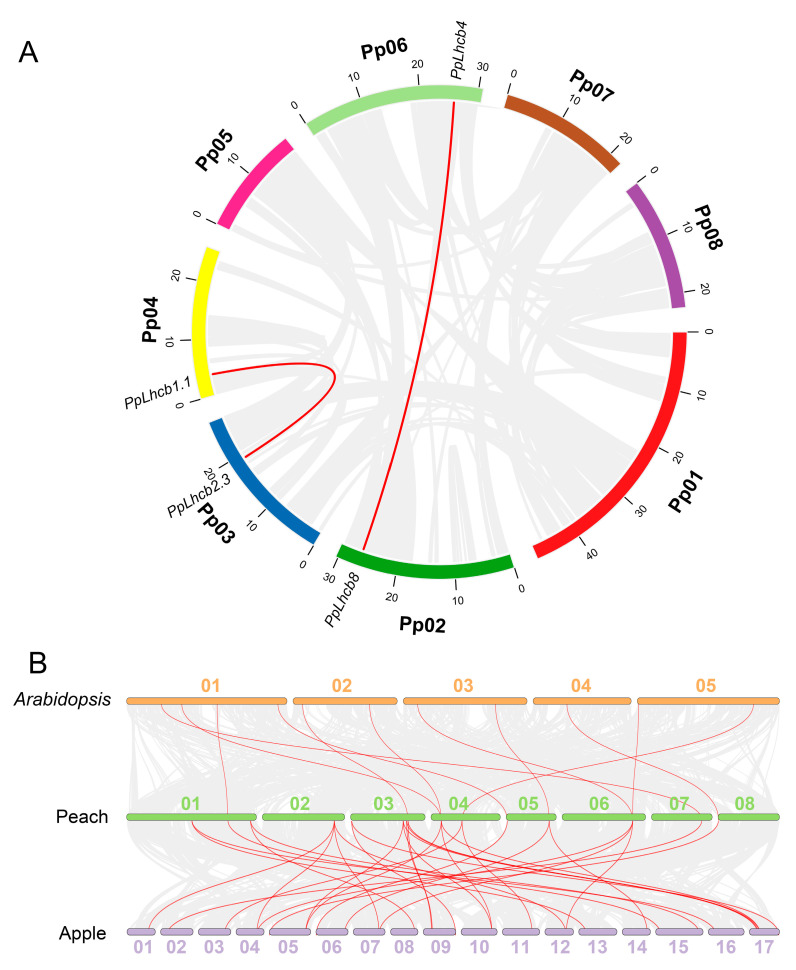
Collinearity analysis. (**A**) Collinearity analysis of the identified 19 *PpLhc* genes in peach genome v2.0.a1. The segmentally duplicated pair is indicated by the red line. (**B**) Large-scale gene collinearity analysis among Arabidopsis, peach, and apple genomes.

To explore the expansion and evolution mechanisms of the *PpLhc* family, gene replication events of the 19 identified *PpLhc* family members were studied, and the results demonstrated that two pairs of homologous genes, namely *PpLhcb1.1*/*PpLhcb2.3* and *PpLhcb4*/*PpLhcb8*, exhibited a collinearity relationship in peach chromosomes (Figure 4A). Previous reports characterized 15 and 27 *Lhc* family members in Arabidopsis and apple, respectively [25]. To gain a deeper understanding of the origin and function of the *PpLhc* gene family, comparative syntenic analysis was conducted among the Arabidopsis, peach, and apple genomes. The results showed that the peach genome had 11 and 31 pairs of collinear *Lhc* genes with Arabidopsis and apple, respectively. In most cases, a single peach *Lhc* gene corresponded to an Arabidopsis *Lhc* gene, which is different from the result where a single peach gene corresponded to multiple apple genes, and vice versa (Figure 4B). These results indicated that peaches were more closely related to apples than to Arabidopsis. We further calculated the Ka/Ks ratio of these synteny genes and found that the Ka/Ks ratio of all gene pairs was much less than 1.0 (Table S3), indicating that most of these *PpLhc* genes were generated after lineage separation and the synteny genes underwent purifying selections.

### 3.6. Identification of Cis-Regulatory Elements in the Promoter of PpLhc Genes

**Figure 5:**
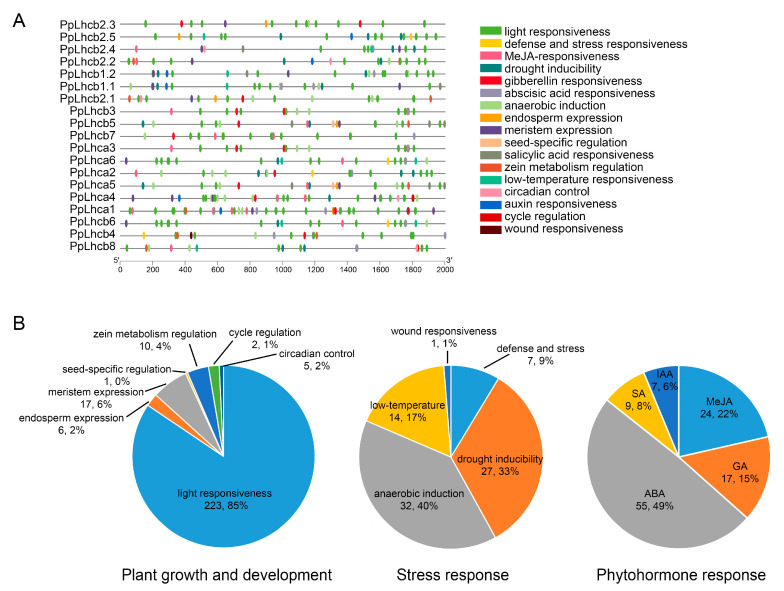
Cis-regulatory elements in the promoters of peach *PpLhc* genes. (**A**) The location of the promoter cis-regulatory elements. (**B**) Distribution of promoter cis-regulatory elements in plant growth and development, stress response, and phytohormone response. Pie chart size represents the proportion of promoter element in each category.

The promoter sequences of 19 *PpLhc* genes, located 2 kb upstream of the 5′ UTR, were analyzed using the PlantCARE database to predict the promoter elements. In addition to the conventional TATA-box and CAAT-box promoter elements, 17 kinds of cis-regulatory elements were identified and found to be widely distributed in these promoters (Figure 5A). These cis-regulatory elements were classified into plant growth and development, stress response, and phytohormone response categories: (Figure 5B). Among the cis-regulatory elements related to plant growth and development, light responsiveness elements accounted for the highest proportion (86%) (Figure 5B), with each member of the peach Lhc family having at least six light responsiveness elements (Table S4), which is in accordance with the important role of the Lhc family in the establishment of light morphology. Among the stress-related elements, anaerobic induction (40%), drought induction (33%), low temperature responsiveness (17%), and defense stress response (9%) were the four main kinds of elements detected (Figure 5B). Only one wound responsiveness element was identified in the promoter region of *PpLhcb4*. The phytohormone response elements in the *PpLhc* gene promoter were mainly related to ABA (49%), MeJA (22%), GA (15%), SA (8%), and IAA (6%) (Figure 5B), indicating that *PpLhc* genes might play important roles in plant growth and development processes in response to various phytohormones. In addition, all *PpLhcs* contained phytohormone and stress response elements except *PpLhcb2.3* (Table S4). These findings suggest that the expression of *PpLhc* genes could be induced by various stressors and plant hormones.

### 3.7. Expression Pattern Analysis of PpLhc Genes under Drought Stress

**Figure 6:**
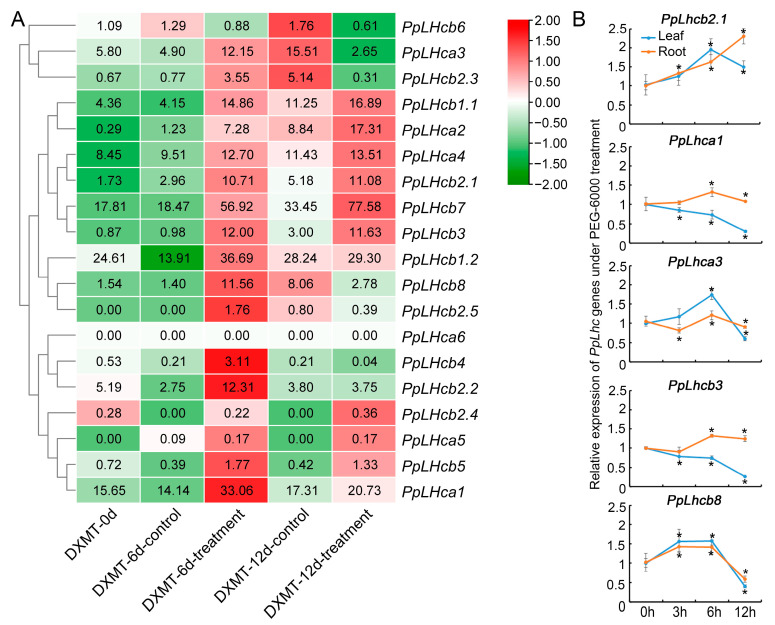
Expression profiles of peach *Lhc* genes in the root and leaf of cv. “Dong Xue Mi Tao” under drought treatment. (**A**) Heatmap analysis of expression patterns of *PpLhc* genes under drought stress at different times. The values presented in the boxes indicate the original TPM values of genes, while the indicator ranges in the upper right corners indicate the range of the values after log normalization. (**B**) Expression analysis of 5 *PpLhcs* under drought stress in peach. The horizontal axis represents hours after PEG-6000 treatment, and the vertical axis represents the expression of *PpLhc* members relative to the internal control gene *PpActin*. The relative expression was calculated with respect to control samples (0 h). The error bars represent ± SE of three replicates. Statistical significance was assessed using two-tailed *t*-tests (* *p* ≤ 0.05).

To investigate the potential function of *PpLhc* genes under drought stress, the expression profiles of peach *Lhc* family genes were examined based on drought transcriptome data downloaded from the NCBI SRA database. A heatmap was constructed based on gene expression levels (TPM values) of 19 *PpLhcs* in the roots of peach cv. “Dong Xue Mi Tao” (DXMT) under drought treatment at different times (Figure 6A). In general, the expression levels of *PpLhc* members could be categorized as high (TPM > 10), medium (2 < TPM < 10), and low (TPM < 2) based on their expression at 0 d treatment. Eleven genes, including *PpLhca3*, *PpLhcb2.3*, *PpLhcb1.1*, *PpLhca2*, *PpLhcb2.1*, *PpLhcb7*, *PpLhcb3*, *PpLhcb1.2*, *PpLhcb8*, *PpLhcb2.2*, and *PpLhca1* were up-regulated at 6 d of drought treatment. Among them, the expression levels of three *PpLhc* genes (*PpLhca3*, *PpLhcb2.3*, and *PpLhcb8*) were downregulated when the drought treatment extended to 12 days, while the remaining eight genes remained upregulated at 12 d of drought treatment. Therefore, it is possible that certain *PpLhc* genes may be involved in regulating the peach’s response to drought stress.

To further confirm the expression patterns of the genes under drought stress at different times, the expression levels of five randomly selected *PpLhcs* in both the root and leaf of peach cv. “Gengcunyangtao” were analyzed using RT–qPCR (Figure 6B). A 20% (*w*/*v*) PEG-6000 solution was used to simulate drought stress. Generally, the RT–qPCR results for root were consistent with the RNA-Seq results mentioned above. For instance, *PpLhcb2.1* was upregulated at different drought times; *PpLhca3* and *PpLhcb8* were upregulated in the early stage of drought stress but downregulated in the later stage (Figure 6B). However, two genes were found to have different expression profiles in root and leaf tissues under drought stress. *PpLhca1* and *PpLhcb3* showed relatively increased expression at different times of stress exposure in root tissue but gradually decreased expression with the extension of drought treatment time. This contradictory result was in line with earlier results in which some family members responded differentially during stress situations in different plant tissues [42,43].

## 4. Discussion

The superfamily of light-harvesting chlorophyll a/b-binding proteins is characterized by the existence of a conserved chlorophyll-binding domain. This superfamily can be divided into four families, namely: Lhc, Lil (light-harvesting-like), PsbS (photosystem II subunit S), and FCII (ferrochelatase II) [6]. Currently, the *Lhc* gene family has been identified in various plant species, including wheat [26], Arabidopsis [44], rice [44], *Cyperus esculentus* [45], pear [46], grape [47], kiwifruit [16], cotton [17,42], papaya [48], pomegranate [49], tea plant [50], cassava [51], potato [52], Populus [8], *Zostera marina* [53], *Gardenia jasminoides* [54], *Ricinus communis* [55], barley [56], and apple [25]. In this study, the first genome-wide characterization of the Lhc family was performed in peach. We identified a total of 19 PpLhc members, which is less than those of two other Rosaceae plants: apple and pear, which have 28 and 27 members, respectively [42,46]. Similar to what is observed in apples and pears, *PpLhc* genes were unevenly distributed on eight chromosomes.

Motif analysis showed that 19 identified PpLhcs contain conserved domains, and Motif 1, Motif 2, and Motif 5 are possessed by most of the PpLhc family members. In addition, members of different subfamilies contain distinct conserved motifs, and members of the same branch of the evolutionary tree have similar ones. These results indicate that the PpLhcs family is highly conserved. In plants, Lhc family members belong to thylakoid membrane proteins, which perform activities such as chlorophyll-binding, pigment-binding, and lipid-binding, which enable them to participate in light absorption and transfer the absorbed energy to the photochemical reaction center. [6]. The results of this study demonstrate that, as predicted, the PpLhc proteins are mainly located in chloroplasts, and all of them have TM domains, suggesting that the PpLhc family members are vital for photosynthesis, which is in line with the results of previous studies [46,51]. The clustering relationship on the evolutionary tree was inferred from the phylogenetic analysis, and as the similarity of gene function increased, the relationship between clusters became closer [57]. In this research, 11 pairs of homologous genes were found between Arabidopsis and peach based on phylogenetic analysis. In general, homologous genes often tend to preserve similar functions throughout the evolution of distinct species [58]. Hence, we speculated that these genes likely possess analogous biological roles.

Gene expression is transcriptionally regulated by the cis-regulatory elements, and the nature of the cis-regulatory element present in the promoter region may also suggest the function of the gene [59]. Thus, to examine the functions of *PpLhc* genes, it is imperative to scrutinize the cis-regulatory elements of their promoters. In this study, 17 kinds of cis elements were predicted to exist in the promoter regions of the *PpLhc* genes, among which, light responsiveness elements are the most abundant ones, existing in all *PpLhc* gene promoters with at least 6 copies, which is consistent with the essential role of Lhc proteins in the plant photosynthetic process, such as that reported for *AtLhcb7* [60], *HvLhcb1* [61], and *ZmLhca* and *ZmLhcb* [53]. Light plays an essential role in plant survival and regulates many biological processes. These light-responsiveness elements indicate that the *PpLhc* family genes function in the process of light-regulated growth and development. Research conducted on different plants has demonstrated that *Lhc* family gene expression is affected not only by light signals, but also by abiotic stress. For instance, oxidative stress could impact *Lhcb* expression [15]. The expression of *TaLHC86* increased after exposure to PEG-6000 and salt and decreased after ABA and SA treatment [26]. Arabidopsis *Lhcb* mutants were found to be more susceptible to drought compared to wild-type plants [22]. These findings suggest that *Lhc* genes play a crucial role in regulating plant resistance to various abiotic stresses. In the case of *PpLhcs*, multiple stress-related elements were discovered in their promoters. It has also been reported that hormone-related elements (ABA, MeJA, SA, GA, and IAA) play important roles in plant responses to plant signal transduction and biotic and abiotic stresses, and these elements were also observed in our research.

The existence of drought-responsive elements in the *PpLhc* promoter regions suggests that some members of this gene family may be involved in the response to drought conditions. By analyzing gene expression through RNA-seq under drought stress, the upregulated *PpLhc* genes could be considered potential candidates for studying drought tolerance in peaches. In addition, the RT–qPCR results indicate that the expression profiles of five randomly selected *PpLhc* family members in root tissue under the mimicked drought stress conditions (20% (*w*/*v*) PEG-6000 treatment) agreed with the heatmap results, further supporting our analysis and prediction. These results suggest that some PpLhc proteins may play vital roles in the drought stress response in peaches, which needs further research.

In conclusion, this study performed a comprehensive genomic survey of *Lhc* genes in peaches. A total of 19 *PpLhc* genes were identified by genome-wide analysis, located on eight chromosomes. The *PpLhc* genes were divided into three subfamilies based on phylogenetic analysis and conserved domain analysis. The promoter regions of the *PpLhc* genes contained a significant number of cis-regulatory elements, indicating that the expression of *PpLhc* genes was controlled by a complex regulatory network. RNA-seq and RT-qPCR analyses revealed that the expression of some *PpLhc* genes was affected under drought stress, suggesting that some *PpLhcs* would be involved in the drought stress response. This study lays the foundation for further investigation into the function of the *PpLhc* gene in peach growth and development and also provides a theoretical basis for the molecular breeding of drought-resistant peach varieties.

## Figures and Tables

**Table 1:** Basic information of *PpLhc* genes in peach. pI = isoelectric point; GRAVY = grand average of hydropathicity.

Gene Name	Locus ID	Chr	Gene Location	Protein Length (AA)	Molecular Weight (kDa)	Theoretical pI	Aliphatic Index	GRAVY
*PpLhca1*	Prupe.8G142700	8	15990280…15992121 forward	247	26.72	6.11	83.36	−0.10
*PpLhca2*	Prupe.1G225700	1	23908446…23910213 reverse	272	29.15	6.42	77.61	−0.05
*PpLhca3*	Prupe.3G191900	3	20429662…20431261 forward	276	29.75	7.85	85.94	−0.05
*PpLhca4*	Prupe.5G195100	5	15839308…15840574 reverse	255	28.17	6.91	86.04	−0.09
*PpLhca5*	Prupe.1G435900	1	37201367…37203304 forward	266	28.84	7.13	97.93	0.15
*PpLhca6*	Prupe.7G195500	7	18520951…18522728 forward	267	29.91	6.12	81.12	−0.10
*PpLhcb1.1*	Prupe.4G076200	4	3725892…3726970 reverse	264	28.03	5.14	79.17	−0.01
*PpLhcb1.2*	Prupe.4G076300	4	3728016…3728975 forward	265	28.13	5.14	78.87	−0.01
*PpLhcb2.1*	Prupe.3G201000	3	20963264…20964787 forward	265	28.48	5.47	81.02	−0.01
*PpLhcb2.2*	Prupe.3G004100	3	271589…272612 forward	265	28.33	5.29	78.11	−0.04
*PpLhcb2.3*	Prupe.3G174600	3	19179155…19179985 reverse	267	28.33	5.3	81.16	−0.02
*PpLhcb2.4*	Prupe.3G174700	3	19181948…19183068 reverse	267	28.33	5.3	81.16	−0.01
*PpLhcb2.5*	Prupe.3G174800	3	19184322…19185343 reverse	208	22.59	5.93	74.62	−0.20
*PpLhcb3*	Prupe.4G190500	4	11367294…11369253 reverse	269	29.12	5.11	84.5	0.02
*PpLhcb4*	Prupe.6G276200	6	25997046…25998631 reverse	286	31.20	5.46	81.61	−0.16
*PpLhcb5*	Prupe.8G001200	8	114709…116781 forward	290	30.92	5.73	90.31	0.00
*PpLhcb6*	Prupe.1G557800	1	45538766…45538766 reverse	259	27.68	7.78	81.12	0.04
*PpLhcb7*	Prupe.5G002700	5	404568…408672 reverse	327	36.16	7.7	101.1	0.09
*PpLhcb8*	Prupe.2G248900	2	26441288…26443733 reverse	282	30.87	5.15	81.31	−0.12

**Table 2:** Subcellular localization and transmembrane prediction of PpLhc proteins.

Protein	Subcellular Localization Prediction			TM Prediction
	SoftBerry	Predotar	WoLF PROST	DeepTMHMM
PpLhcb6	Membrane-bound Chloroplast	Possibly plastid	Chloroplast	3
PpLhca6	Membrane-bound Chloroplast	Possibly plastid	Chloroplast	3
PpLhca2	Membrane-bound Chloroplast	Plastid	Chloroplast	1
PpLhca5	Membrane-bound Chloroplast	none	Chloroplast	3
PpLhcb4	Membrane-bound Chloroplast	Plastid	Chloroplast	3
PpLhcb8	Membrane-bound Chloroplast	Plastid	Plasma membrane	3
PpLhca4	Membrane-bound Chloroplast	Plastid	Chloroplast	3
PpLhca3	Membrane-bound Chloroplast	Plastid	Chloroplast	3
PpLhcb3	Membrane-bound Chloroplast	Plastid	Chloroplast	3
PpLhca1	Membrane-bound Chloroplast	Plastid	Chloroplast	3
PpLhcb2.3	Membrane-bound Chloroplast	Plastid	Chloroplast	3
PpLhcb2.4	Membrane-bound Chloroplast	Plastid	Chloroplast	3
PpLhcb1.2	Membrane-bound Chloroplast	Plastid	Chloroplast	3
PpLhcb1.1	Membrane-bound Chloroplast	Plastid	Chloroplast	3
PpLhcb2.2	Membrane-bound Chloroplast	Plastid	Chloroplast	3
PpLhcb5	Membrane-bound Chloroplast	Plastid	Chloroplast	3
PpLhcb2.1	Membrane-bound Chloroplast	Plastid	Chloroplast	3
PpLhcb7	Membrane-bound Chloroplast	Plastid	Plasma membrane	1
PpLhcb2.5	Membrane-bound Chloroplast	Plastid	Chloroplast	3

## Data Availability

The RNA-seq data used for expression pattern analysis of *PpLhc* genes in response to drought stress was download from NCBI with accession number PRJNA323761.

## Supplementary Materials

The following supporting information can be downloaded at: https://www.mdpi.com/article/10.3390/genes14071475/s1, Table S1: Primers used in this study; Table S2: The sequences of the conserved motifs; Table S3: The synteny *Lhc* genes in *Prunus persica*, *Arabidopsis thaliana*, and *Malus domestica*; Table S4: Variety and number of cis-regulatory elements of each *PpLhc*.
